# Hydrochar from Agricultural Waste as a Biobased Support Matrix Enhances the Bacterial Degradation of Diethyl Phthalate

**DOI:** 10.3390/molecules30051167

**Published:** 2025-03-05

**Authors:** Emanuel Gheorghita Armanu, Simone Bertoldi, Matthias Schmidt, Hermann J. Heipieper, Irina Volf, Christian Eberlein

**Affiliations:** 1 Department of Environmental Engineering and Management, Faculty of Chemical Engineering and Environmental Protection “Cristofor Simionescu”, “Gheorghe Asachi” Technical University, 73A Prof. D. Mangeron Blvd., 700050 Iasi, Romania; gheorghita-emanuel.armanu@student.tuiasi.ro; 2 Department of Molecular Environmental Biotechnology, Helmholtz Centre for Environmental Research (UFZ), 04318 Leipzig, Germany; simone.bertoldi@ufz.de (S.B.); christian.eberlein@ufz.de (C.E.); 3 Department Technical Biogeochemistry, Helmholtz Centre for Environmental Research (UFZ), 04318 Leipzig, Germany; matthias.schmidt@ufz.de

**Keywords:** agricultural biomass valorization, bio-based adsorbent, support matrix, immobilized bacterial cells, plasticizers, microbial biodegradation, sustainable ecotechnologies

## Abstract

The hydrothermal carbonization (HTC) of biomass presents a sustainable approach for waste management and production of value-added materials such as hydrochar, which holds promise as an adsorbent and support matrix for bacterial immobilization applied, e.g., for bioremediation processes of sites contaminated with phthalate ester plasticizers such as diethyl phthalate (DEP). In the present study, hydrochar was synthesized from vine shoots (VSs) biomass employing the following parameters during the HTC process: 260 °C for 30 min with a 1:10 (*w*/*v*) biomass-to-water ratio. The resulting vine shoots hydrochar (VSs-HC) was characterized for porosity, elemental composition, and structural properties using Scanning Electron Microscopy (SEM), Energy-Dispersive X-ray Spectroscopy (EDX), and Raman spectroscopy. Elemental analysis confirmed the presence of key elements in the VSs structure, elements essential for char formation during the HTC process. The VSs-HC exhibited a macroporous structure (>0.5 μm), facilitating diethyl phthalate (DEP) adsorption, bacterial adhesion, and biofilm formation. Adsorption studies showed that the VSs-HC achieved a 90% removal rate for 4 mM DEP within the first hour of contact. Furthermore, VS-HC was tested as a support matrix for a bacterial consortium (*Pseudomonas* spp. and *Microbacterium* sp.) known to degrade DEP. The immobilized bacterial consortium on VSs-HC demonstrated enhanced tolerance to DEP toxicity, degrading 76% of 8 mM DEP within 24 h, compared with 14% by planktonic cultures. This study highlights VSs-HC’s potential as a sustainable and cost-effective material for environmental bioremediation, offering enhanced bacterial cell viability, improved biofilm formation, and efficient plasticizer removal. These findings provide a pathway for mitigating environmental pollution through scalable and low-cost solutions.

## 1. Introduction

**Figure 1:**
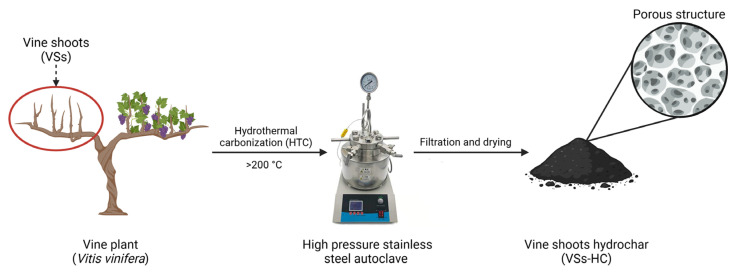
Vine shoot valorization through hydrothermal carbonization and the resulting porous hydrochar.

The global annual generation (>1 billion tons) of large quantities of agricultural waste presents both a challenge and an opportunity for sustainable resource management [1,2]. Hydrothermal carbonization (HTC) has emerged as a promising solution to transform lignocellulosic biomass into hydrochar under moderate temperature and pressure conditions [3]. The resulting material, namely hydrochar, is a carbon-rich material characterized by high porosity and substantial surface area, making it suitable for a variety of environmental applications [4,5]. Among the numerous types of agricultural waste, vine shoots (VSs)—an abundant by-product of vineyard annual pruning—represent an ideal resource for hydrochar production [6]. Re-purposing such biomass aligns with the principles of a circular economy by reducing waste while creating high-value materials for industrial and environmental purposes (Figure 1).

The porous structure and carbon-rich composition of hydrochar offer significant potential in biotechnological applications, particularly as a support matrix for microbial immobilization [7]. In the context of environmental remediation, hydrochar can facilitate the immobilization of bacterial cells, enhancing their stability and efficiency in degrading persistent pollutants, such as phthalate esters [8,9]. Its high surface area and abundant honeycomb structures promote bacterial adhesion and biofilm formation, while its adsorption properties enable it to retain pollutants for microbial degradation [9,10]. Due to its numerous functional groups, hydrochar can easily interact with aromatic benzene-ring-structure pollutants and break them down to less toxic compounds. Such examples of interactions include hydrophobic groups which can enhance adsorption through π–π interactions (stacking) with the benzene ring of DEP; hydroxyl (-OH) and carboxyl (-COOH) groups, which can contribute to hydrogen bonding with the ester (-COO-) groups; and carbonyl (-C=O) groups, which can facilitate dipole–dipole interactions with aromatic molecules [11,12]. These features make hydrochar particularly suited for addressing the contamination of a wide range of pollutants.

In this study, vine shoot hydrochar (VSs-HC) was employed for the removal of diethyl phthalate (DEP), a representative compound of the dialkyl phthalate ester group. Dialkyl phthalate esters, or just phthalate esters, are widely used plasticizers essential for enhancing the flexibility, durability, and processability of materials [13]. Globally, it is estimated that 5 to 8 million tons of phthalate esters are produced each year, of which approximately 1 million tons are potentially used in agricultural plastics, such as mulch films, irrigation tubing, drip lines, seedling trays, packaging, etc. [14]. A portion of these phthalate esters accumulates in waste dumps, contributing to the contamination of agricultural soils. In these soils, the total amount of phthalate esters is estimated to range from 100 to 1000 tons worldwide, corresponding to concentrations of 0.1 to 10 mg/kg [15,16]. Besides agricultural plastics, phthalate esters are commonly found in food packaging, medical devices, flooring, and cosmetics, among other products [17,18]. The increasing demand for these materials has led to the projected growth of the plasticizer market being expected to exceed 14.32 million metric tons by 2030 [19]. However, these benefits come with significant environmental and health concerns. Phthalate esters are non-covalently bound to polymer matrices and gradually leach into the environment. This makes them one of the most frequently detected persistent organic pollutants [20]. Their chemical toxicity disrupts biological membranes, and as endocrine disruptors, they pose serious risks to human health [21]. Phthalate esters are typical amphipathic molecules with a high hydrophobicity as well as a relatively good water solubility. These molecules show a tendency to dissolve relatively well in the phospholipid bilayer of biological membranes [22,23]. The accumulation of these amphipathic organic compounds leads to a non-specific increase in membrane fluidity causing permeabilization of the cell membranes that causes damage to cell metabolism [24,25]. Consequently, phthalate esters, as typical aromatic structures with hydrophilic substituents, are known to be among the most toxic substances, making them very hazardous to the environment but also toxic to bacteria capable of degrading them [22,26].

Given these challenges, addressing phthalate ester contamination has become an environmental priority. Traditional remediation methods, such as chemical treatments and physical removal, often fall short due to high costs, incomplete degradation, and the risk of secondary pollution [27]. Bioremediation has emerged as a promising alternative, leveraging microorganisms’ metabolic pathways to degrade pollutants into less harmful by-products or mineralize them [28]. Several bacterial strains of the genera *Pseudomonas*, *Bacillus*, *Microbacterium*, and *Rhodococcus* have been reported to effectively biodegrade phthalate esters through enzymatic hydrolysis and oxidative degradation (monooxygenases and dioxygenases) [29,30,31,32]. Both mechanisms are complementary, and they often work together in microbial degradation pathways. Despite its potential, the inherent toxicity of DEP often reduces the efficiency of free-living bacterial cells in such processes, creating a need for innovative strategies to enhance bioremediation. One such strategy involves immobilizing bacteria on suitable support materials to improve cell stability, biofilm formation, and tolerance to toxic pollutants [33].

In this study, VSs-HC was employed as a support material for immobilizing a bacterial consortium to enhance the biodegradation of DEP. DEP was selected as a model compound due to its environmental persistence and toxicity. HTC was used to produce hydrochar from VSs biomass, and its properties were characterized using techniques such as Scanning Electron Microscopy (SEM), Raman spectroscopy, Energy-Dispersive X-ray Spectroscopy (EDX), and Ultra-Performance Liquid Chromatography (UPLC). These analyses confirmed the hydrochar’s porous structure, composition, and ability to support efficient bacterial immobilization. It is commonly known that immobilized bacterial cells exhibit improved tolerance and stability, effectively degrading high concentrations of toxic organic compounds such as DEP [23,34,35]. By promoting hydrochar as a bio-based solution for addressing phthalate contamination, this study demonstrates a sustainable approach that integrates waste valorization with effective environmental remediation.

## 2. Results and Discussions

### 2.1. Hydrochar Preparation and Characterization

#### 2.1.1. VSs Conversion Through HTC

**Figure 2:**
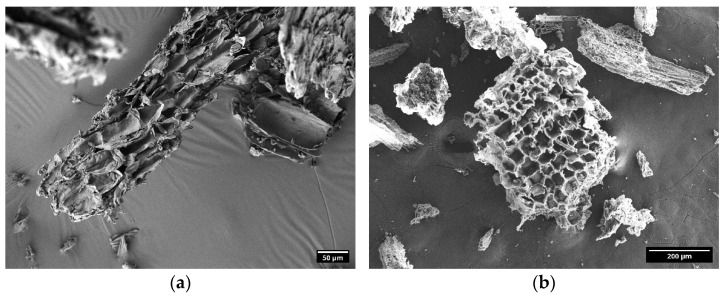
SEM images of (**a**) VSs biomass; and (**b**) VSs-HC obtained at 260 °C, 30 min, and 1:10 *w*/*v* ratio.

During the HTC process, the raw material structure underwent significant physical transformations and a new structure was shaped. A honeycomb-like structure was revealed under SEM, suggesting that the HTC process effectively decomposed the lignocellulosic structures and improved the pore formation (Figure 2). Compared to the VSs (Figure 2a) biomass, which exhibited rough and heterogeneous fiber structures, the resulting hydrochar (Figure 2b) exhibited well-defined porous structures with diameters varying from 0.5 up to 20 μm, as observed under SEM. According to the IUPAC classification for porous materials [36], the obtained hydrochar contains large and observable macropores (>50 nm).

#### 2.1.2. SEM-EDX Analysis

**Figure 3:**
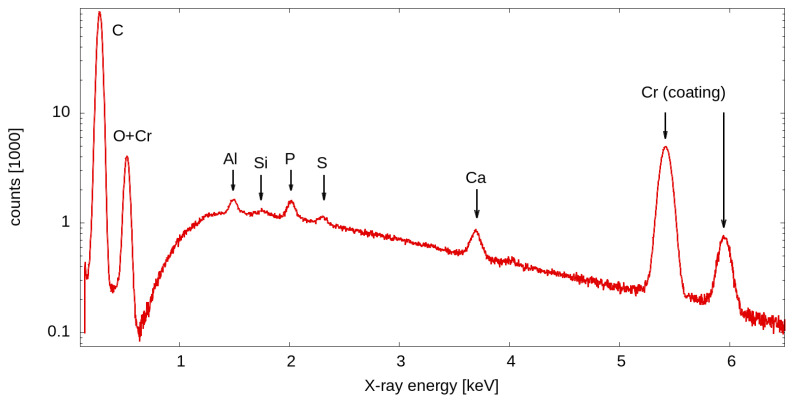
SEM-EDX coupled analysis of the hydrochar surface.

Elemental analysis (SEM-EDX) revealed that besides the main constituent carbon (C), hydrochar exhibited comparatively high levels of other elements such as calcium (Ca), oxygen (O), aluminum (Al), silicon (Si), phosphorous (P), and sulphur (S). The presence of calcium (Ca) and silicon (Si) is particularly important, as these elements contribute to the structural stability of hydrochar and influence its thermal degradation behavior and adsorption properties (Figure 3) [37]. From the perspective of microbial immobilization, the carbon-rich surface of hydrochar provides hydrophobic interactions that enhance bacterial attachment. Oxygen facilitates hydrogen bonding and electrostatic interactions, improving bacterial adhesion on the pore structures [38]. Elements such as Ca, P, S, and Si promote biofilm formation by stabilizing extracellular polymeric substances (EPSs) secreted by bacteria, thereby increasing bacterial adhesion and biofilm stability and enhancing pollutant resistance [39]. More than that, due to its divalent nature, Ca acts as a bridging agent between negatively charged bacterial cells and hydrochar surfaces, facilitating bacterial adhesion [40]. Meanwhile, the high levels of chromium (Cr) observed in the SEM-EDX analysis result from the sputter-coated layer (30 nm) of the hydrochar sample and are not a constituent or inherent part of the VSs biomass.

#### 2.1.3. Raman Spectroscopy of VSs-HC

**Figure 4:**
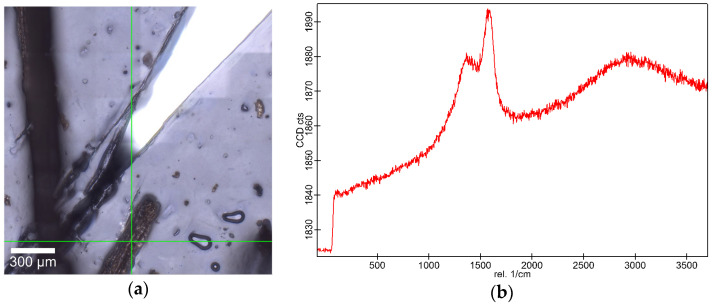
Hydrochar’s Raman analysis on (**a**) targeted location and (**b**) the identified spectral band of graphite-like structures.

Understanding the structural composition of biomass is crucial for optimizing materials for specific applications, such as microbial immobilization. Raman spectroscopy was employed to analyze the structural features of VSs-HC using a WITec Raman microscope (Figure 4). The spectra revealed distinct peaks at 1370 cm^−1^ and 1583 cm^−1^, corresponding to the D- and G-bands of graphite-like structures, which are indicative of carbonization.

The aforementioned Raman shifts suggest the formation of disordered and graphitic carbon, which has significant implications for microbial immobilization. The presence of graphitic domains enhances electrical conductivity, a critical parameter for electron transfer in bioelectrochemical applications [41]. Additionally, carbonaceous structures, such as hydrochar, provide a favorable surface for microbial adhesion due to their π–electron interactions and the potential presence of oxygen-containing functional groups [42].

Although cellulose and hemicellulose play vital roles in hydrothermal carbonization (HTC) by influencing pore formation and surface chemistry, their identification cannot be confirmed based on the Raman spectra obtained. This means that both biopolymers were broken down during the HTC process into smaller organic compounds, such as 5-hydroxymethylfurfural (HMF), furfural, levulinic acid, and various phenolic derivatives, compounds which do not retain the crystalline structure required for characteristic Raman signals [43]. Additionally, the HTC process promotes the dehydration, decarboxylation, and aromatization of the VSs-HC with the formation of hydrochar, which consists of disordered carbon, oxygen-containing functional groups, and aromatic domains [44]. Therefore, the decomposition of cellulose and hemicellulose lead to new reorganized chemical structures dominated by aromatic and disordered carbon species. As a result, their characteristic Raman spectra disappear, replaced by broad D- and G-bands typical of hydrochar. Nevertheless, the new graphene-like structures are more likely to improve surface properties for microbial attachment and electron transfer processes.

### 2.2. Adsorption of DEP by Hydrochar

**Figure 5:**
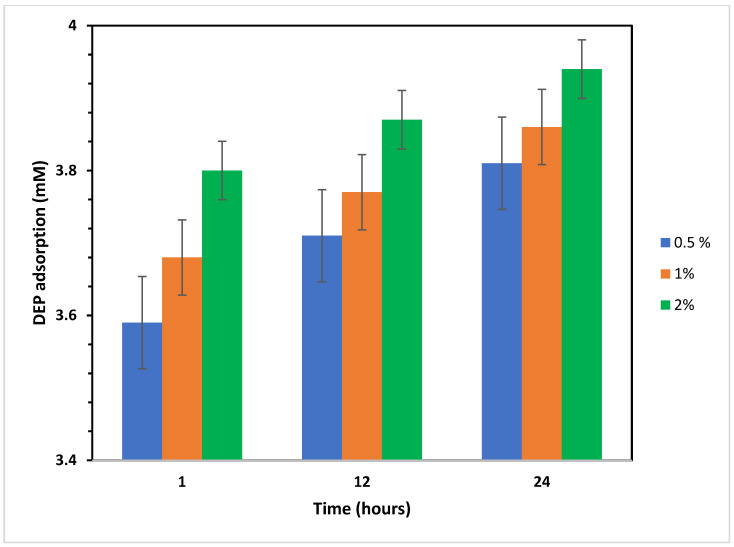
UPLC measurements of DEP adsorption by different amendments of hydrochar after 1 h (T1), 12 h (T12), and 24 h (T24). The initial concentration was 4 mM DEP. Hydrochar concentrations (%) reported for the dry weights (mg) of 0.5 = 50 (blue); 1 = 100 (orange); 2 = 200 (green).

The adsorption efficiency of hydrochar was evident from the rapid reduction in DEP concentration. Within the first hour of contact, 3.59 mM DEP (90%) was adsorbed from 4 mM DEP by using 0.5% (50 mg) VSs-HC, as shown in Figure 5. The DEP adsorption increased gradually over time, and after 24 h, approximately 3.81 mM (95%) of DEP was removed in experiments using only 0.5% VSs-HC.

Physicochemical interactions and diffusion processes can explain the mechanisms behind the rapid adsorption (initial phase) and slow adsorption (later phase) of DEP by hydrochar. The porous structure of hydrochar facilitates DEP diffusing into the available pores and binding to active sites (mainly functional groups and surface area). However, as these sites fill up, the process slows down and the available DEP must diffuse deeper into smaller pores or through the hydrochar matrix, which takes longer. Additionally, hydrochar contains various oxygen-containing functional groups (e.g., hydroxyl (-OH), carboxyl (-COOH), and carbonyl (-C=O)), which interact with DEP via hydrogen bonds, electrostatic attraction, or π-π interactions [45]. Over time, as these pores become occupied, the adsorption rate decreases as observed at 12 and 24 h.

Although 2% of VSs-HC (~98% after 24 h) adsorbed more DEP than 0.5% of VSs-HC (~95% after 24 h), a major difference was not observed and further tests were continued with the lowest concentration of hydrochar. Therefore, a hydrochar concentration of 0.5% was identified as optimal for future experimental applications, ensuring both high adsorption capacity and material efficiency.

### 2.3. Microbial DEP Removal in the Presence and Absence of Hydrochar

**Figure 6:**
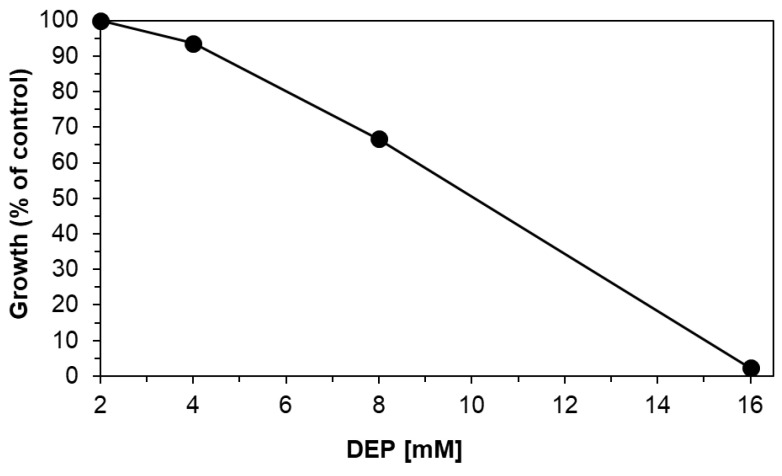
Growth rates (black circles) of the bacterial consortium when incubated with different concentrations of DEP in the absence of hydrochar. The growth rate of the bacterial consortium on 2 mM DEP (=control) was set to 100% and compared to the growth rates on 4, 8, and 16 mM DEP. Growth on 32 mM DEP is 0% of the control.

The removal of DEP by a microbial consortium, mainly consisting of *Pseudomonas* spp. and *Microbacterium* sp., was tested in the presence and absence of VSs-HC. Figure 6 shows the growth rates of the bacterial consortium in the absence of hydrochar on different concentrations of DEP. The growth rates of 4, 8, 16, and 32 mM were compared to the that of 2 mM, with the latter serving as a control. Although the amount of carbon and energy increased with the addition of 4 mM DEP, the growth rate decreased by about 6%. The toxic effects of DEP became much more pronounced when adding 8 mM, resulting in growth rates of 67% compared to the control. When adding 16 mM DEP, strong inhibitory effects were observed; the bacterial consortium barely survived with a 2% growth compared with 2 mM DEP (control).

**Figure 7:**
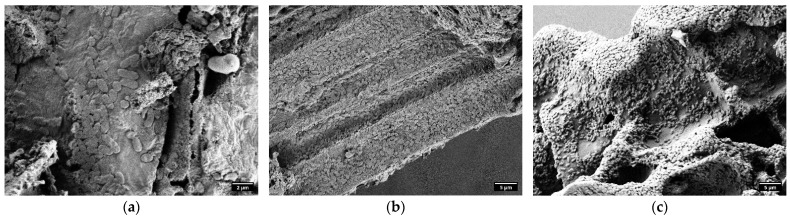
SEM images of VSs-HC after incubation with the bacterial consortium at 8 mM DEP after 1 (**a**), 12 (**b**), and 24 h (**c**).

On the electron micrograph samples (Figure 7), large biofilms can be observed at 8 mM and 16 mM DEP. The observed bacteria present rod shapes and a single flagellum (0.5 to 1 μm in length). These findings confirm that VSs-HC serves as a suitable support matrix for the bacterial cell adhesion and biofilm formation, although at 32 mM DEP, the samples presented lysed cells and scattered fragments of cell membrane.

**Figure 8:**
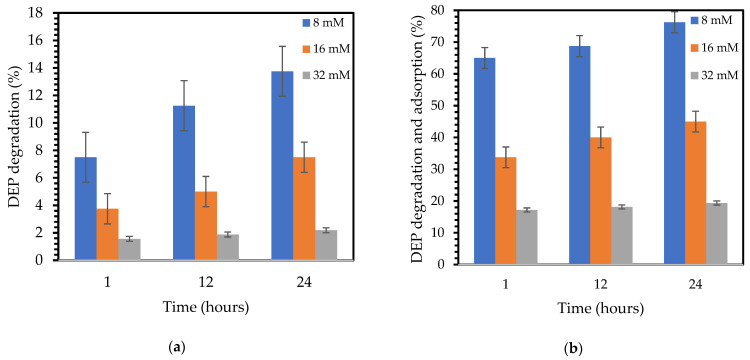
DEP degradation (**a**) by the microbial consortium in the absence of hydrochar and (**b**) by the microbial consortium and hydrochar (0.5% *w*/*v*). Adsorption capacity is reported in mM to reflect the molar concentration of DEP removed from the aqueous solution, consistent with microbiological conventions for substrate measurement. This unit emphasizes the integrated adsorption–biodegradation process rather than hydrochar-specific capacity, which could alternatively be expressed in mg/g or mM/g if normalized to adsorbent mass.

Figure 8 shows that DEP degradation was lower in the planktonic cell culture (Figure 8a) compared to the immobilized cells on the VSs-HC (Figure 8b). The planktonic cells were partially inhibited at 8 and 16 mM DEP, exhibiting a removal of 11% and 5% in the first 12 h of contact, while at 32 mM DEP the bacterial cells are nearly completely inhibited and no activity is observed. The residual 2% DEP degradation measured in this culture (Figure 8a) was found to remain constant during the experiment, which can be attributed to the sorption by necrotic bacterial biomass into cellular structures (like membranes and proteins) [46].

A synergic mechanism of adsorption, by the VSs-HC, and degradation, by the bacterial cells, can be observed. A similar example of combined adsorption and biodegradation processes was found for the removal of the pesticide chlorpyrifos from water by using rice husk biochar and an immobilized bacterial strain of *Aeromonas veronii*. The immobilized bacteria could degrade up to 96% of the chlorpyrifos (30 mg/L) within 24 h, compared with 67% in the planktonic cells [47].

During the first hour of contact, around 65% of the 8 mM DEP was adsorbed and degraded by the VSs-HC and immobilized cells. For 16 mM and 32 mM DEP, the adsorption and degradation of DEP increased by up to 45% and 19% within 24 h of contact. Thus, the adsorption capacity for 8, 16, and 32 mM with 0.5% (*v*/*w*) hydrochar is high enough to ease the toxic effect caused by these high DEP concentrations on the bacterial consortium and facilitate their growth and biofilm formation. However, 32 mM showed a negligible growth, and within 24 h the bacterial cells were hardly inhibited and the possible synergy can be attributed to the adsorption and sorption (by the necrotic bacterial biomass) processes.

The solubility of DEP (1.08 g/L, equivalent to approximately 5.15 mM) indicates that the concentrations used in this study exceeded the compound’s natural solubility, potentially leading to localized aggregation such as in Figure 7. Notably, the entrapped DEP within the hydrochar’s porous structure could serve as a sustained-release mechanism, enabling prolonged antimicrobial activity while minimizing free DEP concentration in the medium. This entrapment likely mitigates the environmental toxicity of DEP while enhancing its bioavailability and efficacy against microbial communities.

## 3. Materials and Methods

### 3.1. Feedstock

One type of raw material was collected from the annual vine pruning of vineyards, namely VSs, and subjected to analysis. The aforementioned raw material was sourced from northeastern Romania and collected during the autumn season. Firstly, it was air-dried in a ventilated room to minimize photodegradation and alteration. Secondly, the material was ground using a Micro Powder Grinding Mill (Retsch GmbH GM 200, Haan, Germany) at a rotation speed of 320 rpm for 20 min to achieve an approximately uniform particle size. Milled material was subsequently classified by particle size using a Retsch GmbH AS 200 sieve shaker (Haan, Germany), separating it into three distinct fractions: large (1–2 cm), medium (0.5–1 cm), and fine (<0.1 cm). The resulting fractions were collected in a box and stored in a desiccator to prevent moisture absorption until further analysis.

### 3.2. Hydrochar Preparation

The HTC of VSs was realized by using a micro pressured stainless-steel autoclave, containing a glass vessel with a volume of 15 mL and a maximum pressure of 10 MPa. For each run, 1 g of VSs was introduced to 10 mL sterile distilled water in order to reach the 1:10 (*w*/*v*) ratio and heated at 260 °C for 30 min. Prior to HTC, the mixture was left to “sink” for 30 min in order to assure a better homogeneity. During the HTC process, the autoclave’s pressure reached approximately 4.5 MPa. After the HTC process was completed, the stainless-steel autoclave was cooled down at room temperature; when it reached 100 °C, it was partially introduced into an ice bath. This procedure sped up the cooling process and provided sufficient time for the autoclaves to depressurize.

The wet hydrochar was filtered through Whatman filter paper (11 μm pore size) in order to separate the dry material from the liquid phase. The obtained solid phase was washed with approximately 50 mL deionized water until the yellowish coloration of the material was no longer present. The liquid phase was collected in 50 mL Duran laboratory bottles and stored in the fridge for further analysis.

The filtered hydrochar was dried at 105 ± 2 °C for 12 h, and then it was weighted until values were constant and stored at room temperature in a closed glass jar for further characterization. For significant data accuracy and reproducibility, all experiments were performed in triplicate.

### 3.3. Preparation of VSs-HC Samples Incubated with the Bacterial Consortium

Approximately 50 mg of VSs-HC was introduced to a 50 mL Erlenmeyer flask and sterilized for 15 min at 121 °C in order to eliminate any native microbial activity. Experiments were performed in triplicates, and therefore 9 Erlenmeyer flasks containing sterilized VSs-HC were prepared.

The sterilized VSs-HC (50 mg), mineral media (10 mL), bacterial consortium inoculum (500 µL), and DEP (8, 16, and 32 mM) were mixed all together in 50 mL Erlenmeyer flasks at 30 °C and 150 rpm for 24 h. During the incubation time, regular sampling (1 h, 12 h, and 24 h) was performed for UPLC analysis. Approximately 0.5 mL aliquots were taken using Whatman filter syringes (Whatman, Darmstadt, Germany; 0.45 μm pore size). The remaining VSs-HC samples were placed on sterile Petri dishes and carefully dried under a horizontal laminar flow hood for 12 h. After drying, samples were transferred to Eppendorf tubes and labeled for further SEM analysis.

Prior to the bacterial immobilization on VSs-HC, an overnight culture was prepared as a stock culture for the further inoculations. The bacterial consortium was cultivated in a 150 mL Erlenmeyer flask containing 50 mL mineral medium and 200 mg/L yeast extract. The overnight culture was kept for a minimum of 8 h at 30 °C and 150 rpm in order to ensure optimal microbial growth before the immobilization process.

### 3.4. Scanning Electron Microscopy (SEM) Analysis, EDX Coupled

Field emission electron microscopy (FE-SEM, Zeiss Merlin VP Compact, Carl Zeiss Microscopy, Oberkochen, Germany) was employed to obtain microscopic information on the morphology and structure of the sample surface. In preparation for SEM, the samples had to be fixed, dehydrated, and dried in order to make them compatible with the high-vacuum conditions in the analysis chamber of the FE-SEM. For that, the samples were chemically fixed in a 2.5% glutaraldehyde solution for 12 h in the fridge, dehydrated in a graded ethanol/water series (30%, 50%, 70%, 80%, 90%, 95%, 100%), subjected to the chemical drying agent hexamethyldisilazane (HMDS, 1:1 HMDS/ethanol, and pure HMDS for 10 min each) and finally air-dried under a fume hood. The fixed and dried samples were then mounted onto standard SEM sample holders (stubs) and were sputter-coated with a 30 nm thick layer of chromium by a Leica SCD500 (Leica Microsystems, Wetzlar, Germany). Imaging was conducted at an electron acceleration voltage of 2 kV with a beam current of approximately 250 pA to 300 pA in secondary electron detection mode. The elemental composition of the sample was obtained by an Energy-Dispersive X-ray (EDX) Spectrometer (Bruker Quantax XFlash 5060F, Bruker Nano GmbH, Berlin, Germany) which is coupled to the FE-SEM. In order to achieve the efficient ionization of chemical elements (heavier than carbon) in the sample, an electron energy of 10 keV was used. Spectra were obtained and evaluated with the software package Bruker Esprit Version 1.9 (Bruker Nano GmbH, Berlin, Germany).

### 3.5. Raman Spectroscopy

For the analysis of the degree of carbonization of VSs-HC, a WITec Alpha 300RA confocal Raman microscope (WITec—Wissenschaftliche Instrumente und Technologie GmbH, Ulm, Germany) was used. Raman spectra were acquired on chunks (<1 mm) of the VSs-HC using a solid-state laser for excitation with a wavelength of 532 nm. Raman spectral acquisition focused on the D- and G-bands of graphite centered at 1370 cm^−1^ and 1583 cm^−1^, respectively [48].

### 3.6. Chemicals

The diethyl phthalate (DEP) of 99% purity used in this study was acquired from Thermo Fisher Scientific (Waltham, MA, USA). Other chemicals and solvents utilized throughout the experiments were of analytical grade, ensuring high purity suitable for accurate and reproducible results. All compounds were utilized as received without any additional purification, maintaining consistency in experimental conditions. This approach minimizes variability and aligns with standard practices for chemical analysis and environmental remediation studies.

### 3.7. Isolation and Cultivation of Bacterial Consortium

A bacterial consortium mainly consisting of *Pseudomonas* spp. and *Microbacterium* sp. capable of efficiently mineralizing different phthalate esters was previously isolated from a biofilm scratch taken from a polyurethane tubing (obtained from the lab of Hermann J. Heipieper). The microbial consortium was cultivated in a mineral medium as described by Hartmans [49] with 200 mg/L yeast extract at 30 °C and 180 rpm in 50 mL scale. As the main carbon and energy source, DEP was added in different concentrations (2, 4, 8, 16, and 32 mM). Growth inhibition (Formula (2)) observed at concentrations higher than 2 mM DEP was calculated by comparing the percentage difference in the growth rates µ (h^−1^) (Formula (1)) between cultures containing more than 4 mM DEP and higher (µ_xmM_) with that of the culture grown with 2 mM DEP (µ_2mM_).

Formula (1): Calculation of bacterial growth rates μ (h^−1^)

μ (h^−1^) = [ln(OD_t1_) − ln(OD_t0_)]/(t_1_ − t_0_) (1)

Formula (2): Relative growth (%)

Relative growth (%) = [μ_xmM_ × 100]/μ_2mM_ (2)

Note: OD represents optical density, a measure of bacterial concentration typically determined by absorbance at a specific wavelength (e.g., 560 nm). OD_t1_ and OD_t0_ denote optical density at times t_1_ and t_0_, respectively.

In addition to the above-described conditions, 10 mL cultures were incubated with 0.5, 1, and 2% (*v*/*w*) VSs-HC in a separate experimental batch at 4 mM DEP. For 0.5% (*v*/*w*) hydrochar, this was also performed at 8, 16, and 32 mM. VSs-HC samples containing DEP were filtered using Whatman filter syringes (0.45 μm pore size) and the resulting aliquots (0.5 mL) were collected in 1.5 mL Eppendorf tubes and stored at −20 °C for further analysis. During the whole sampling and filtering process, Erlenmeyer flasks were kept at 30 °C and 150 rpm, while being collected at the corresponding times (1, 12, and 24 h). The experiments were performed in triplicate.

### 3.8. Analysis of DEP by Ultra-Performance Liquid Chromatography (UPLC)

From the cultures containing the bacterial consortium, different concentrations of DEP samples (0.5 mL) were taken with Whatman filter syringes (0.45 μm pore size), placed in 1.5 mL Eppendorf tubes, and stored at −20 °C until further use. Before analysis, samples were thawed at room temperature, supplemented with 0.5 mL methanol, and shaken at 150 rpm for 5 min (Vortex-Genie 2, Scientific Industries, New York, NY, USA). Aliquots (2.0 μL) of the resulting mixture were directly subjected to measurements. UPLC analysis was performed using an Aquity^TM^ UPLC system (Waters, Eschborn, Germany) equipped with an Aquity^TM^ UPLC BEH C18 column (1.7 µM particle size, 2.1 × 50 mm; Waters, Eschborn, Germany) as described before by Hofmann and Schlosser [50]. Also, the Acquity^TM^ UPLC system was coupled to a PAD detector (absorbance at 278 nm; Waters, Eschborn, Germany) and Empower 3 software. The measurements were performed in isocratic elution at a flow rate of 0.5 mL × min^−1^. Elution was performed with a 50:50 (*v*/*v*) mixture of methanol/water.

## 4. Conclusions

VSs-HC produced through HTC at 260 °C for 30 min at a 1:10 *w*/*v* ratio exhibited remarkable potential, firstly as an excellent adsorptive material and secondly as a natural support matrix for the immobilization of a bacterial consortium. SEM analysis confirmed the presence of macropores (>0.5 μm in diameter) on the VSs-HC, which provided a suitable adhesion surface for bacterial colonization and biofilm formation. Rich in elements such as C, Ca, O, Si, Al, etc., it can be easily converted through HTC to obtain a porous structure.

Raman spectroscopy confirmed the presence of D- and G-bands of graphite-like structures, indicative of carbonization. The HTC process breaks cellulose and hemicellulose into smaller organic compounds and reshapes the chemical structure of hydrochar, creating new carbon structures. Nevertheless, this structural modification leads to improvements in functional groups, microbial adhesion, and electron transfer processes.

VSs-HC’s adsorption properties enable rapid DEP adsorption, with 90% adsorption in just 1 h of contact. Furthermore, in the experiments with planktonic and immobilized bacterial cells, major differences were observed. At 8 mM and 16 mM DEP, hydrochar provides a stable and protective environment for biofilm formation with rates of 65% and 34% DEP degradation, while planktonic cells show 8% and 4% DEP degradation in 1 h. In the immobilized bacterial cells, a maximum of 76% DEP removal was measured with 8 mM after 24 h compared with 14% in the planktonic cells. The DEP-degrading bacterial consortium remains viable within the VSs-HC matrix. A synergic mechanism of adsorption and degradation can be noticed between VSs-HC and the bacterial consortium.

These findings highlight the potential of VSs-HC as a sustainable biosorbent and support matrix for bacterial cells. Therefore, VSs biomass valorization through HTC can be a proper solution for mitigating the environmental impact of organic pollutants such as DEP and valorizing agricultural waste.

## Data Availability

The original contributions presented in the study are partially included in the article, and further inquiries can be directed to the corresponding authors.
